# Early outcomes of robotic management of aberrant subclavian artery

**DOI:** 10.1016/j.xjtc.2025.04.026

**Published:** 2025-05-15

**Authors:** Bo Chang Brian Wu, Meng-Han Tsai, Nicolas Chanes, Drake S. Rosenberg, Robert A. Meguid, Muhammad Aftab, T. Brett Reece, John D. Mitchell

**Affiliations:** a Department of Surgery, University of Colorado Anschutz Medical Campus, Aurora, Colo; b Division of Cardiothoracic Surgery, Department of Surgery, University of Colorado Anschutz Medical Campus, Aurora, Colo

**Keywords:** aberrant subclavian artery, aberrant right subclavian artery, aberrant left subclavian artery, Kommerell diverticulum, minimally invasive, robotic

## Abstract

**Background:**

Aberrant subclavian artery (ASA), though rare, can cause dysphagia lusoria and significantly affect quality of life. Conventional treatment involves open ligation and division of ASA, but a robotic approach is becoming more popular. This study assessed outcomes in patients undergoing robotic ASA division.

**Methods:**

We retrospectively reviewed 9 patients with dysphagia who underwent robotic division of ASA between 2021 and 2025. Our standard approach is one-stage robotic ligation and division of the ASA, followed by open subclavian-to-carotid transposition (SCT). Patients undergo continued surveillance for potential thoracic endovascular aortic repair (TEVAR) in the event that Kommerell diverticulum (KD) expands. Patient demographics, presentations, aberrant anatomy, operative details, and outcomes were reviewed.

**Results:**

The study cohort had a mean age of 49 years and mean body mass index of 30.5 kg/m^2^. The main presenting symptoms were dysphagia and dyspnea. An aberrant left subclavian artery with right-sided arch was seen in 5 patients; an aberrant right subclavian artery, in 4. Seven patients had KD. Eight patients underwent concurrent SCT; 1 patient had prior TEVAR and carotid-subclavian bypass for large descending thoracic and Kommerell aneurysms. The mean operative time was 169 minutes, and the mean hospital stay was 2 days. There was no postoperative stroke, bleeding, pneumothorax, chyle leak, or mortality; 1 patient experienced transient Horner syndrome. Five patients reported significant improvement in dysphagia, 1 reported moderate improvement, 1 reported mild improvement, 1 reported no change, and 1 was lost to follow-up.

**Conclusions:**

This one-stage hybrid approach—robotic ASA division with open SCT—is safe, with no reported postoperative stroke or mortality, and offers excellent patient satisfaction. It provides a minimally invasive alternative for treating dysphagia lusoria.

**Figure:**
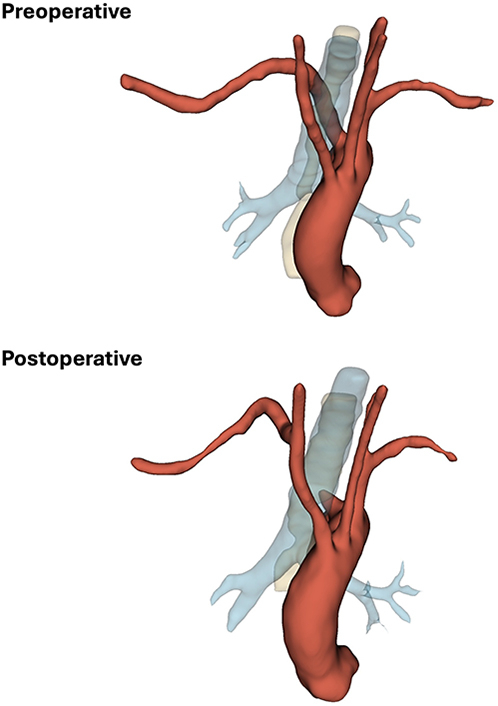
Aberrant subclavian artery before and after robotic division with open transposition.

**Central Message**

Robotic aberrant subclavian artery division with supraclavicular subclavian-to-carotid transposition is a safe, minimally invasive treatment for dysphagia lusoria, with no postoperative stroke or mortality.

**Perspective**

The combined robotic division of the aberrant subclavian artery with supraclavicular subclavian-to-carotid transposition represents a significant advancement in the treatment of dysphagia lusoria. This approach offers excellent symptom relief and patient satisfaction with low morbidity. Long-term surveillance is needed to assess durability and potential aneurysm progression.

Aberrant subclavian artery (ASA) is the most common congenital anomaly of the aortic arch, with a prevalence of 0.5% to 2% in the general population, and is sometimes associated with Kommerell diverticulum (KD), a dilation at the origin of the ASA.^1^ In normal anatomy, the aortic arch has 3 branches: the innominate, left common carotid, and and left subclavian arteries. In the aberrant setting, the aberrant right subclavian artery (ARSA) (Figure 1), the most common variant of ASA, comes off the aortic arch distal to the origin of the left subclavian artery, separate from the right common carotid artery. It typically passes to the right side in a retroesophageal fashion, although there are reports of interesophageotracheal and pretracheal pathways.^2^ The ARSA has been described as “arteria lusoria”, ^3^ and the condition of esophageal compression caused by ARSA is termed “dysphagia lusoria”.^4^ The less prevalent variant of ASA is aberrant left subclavian artery (ALSA) with a right-sided aortic arch (Figure 2).

**Figure 1:**
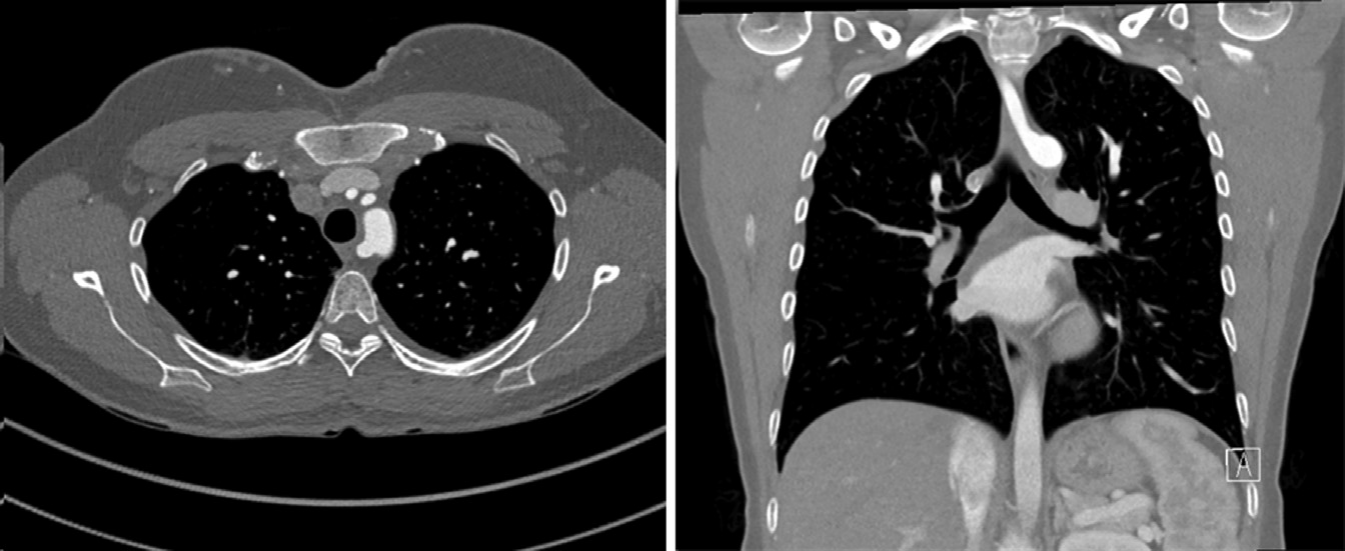
Contrast-enhanced computed tomography scan images illustrating the retroesophageal course of aberrant right subclavian artery. *Left*, axial view; *right*, coronal view.

**Figure 2:**
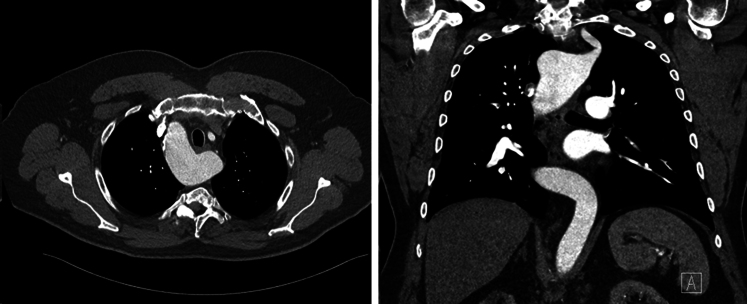
Contrast-enhanced computed tomography scan images illustrating the retroesophageal course of aberrant left subclavian artery with a right-sided aortic arch. Kommerell diverticulum is present. *Left*, axial view; *right*, coronal view.

ASA typically has an indolent presentation, with up to 10% of patients presenting with compressive symptoms.^1^^,^^5^ The most common presentation is dysphagia and shortness of breath^5^; other symptoms include retrosternal chest pain, chronic cough, pulmonary infection, and weight loss.^5^^,^^6^ Although rare, dysphagia lusoria can significantly affect quality of life. Current European Association for Cardiothoracic Surgery/Society of Thoracic Surgeons guidelines recommend surgical intervention for patients with symptomatic ASA to release the compression.^6^ Open surgery via a thoracotomy and carotid-subclavian bypass is recommended for young patients without significant comorbidities.^6^ In contrast, an endovascular, hybrid approach is reserved for patients with an emergency presentation or those who might not tolerate open repair.^6^

The University of Colorado team previously reported success using video-assisted thoracoscopic surgery (VATS) for division of the ASA, followed by concomitant subclavian-to-carotid transposition (SCT).^7^ Building on this technical success, the same team further explored the feasibility of using a robotic approach to replace conventional thoracotomy and VATS. During the same period, few cases were reported describing the use of a robotic approach in managing ASA and KD.^8^^,^^9^

This study aimed to evaluate the early outcomes of patients undergoing robotic surgery for ASA with or without KD at a high-volume aortic center.

## Methods

### Study Design and Patient Selection

This descriptive retrospective study analyzing a single-institution database and electronic medical records was approved by the Colorado Multiple Institutional Review Board (COMIRB #17-0198; approved February 6, 2017). The need for informed written consent for data publication was waived. Between 2017 and January 2025, 15 patients presenting with ASA, with or without KD, who were referred to the cardiothoracic surgery clinic and underwent elective surgical repair of ASA were identified. Among these, 9 symptomatic patients who underwent robot-assisted division of ASA between 2021 and 2025 were included in the study, after excluding 2 asymptomatic patients receiving surgery in preparation for frozen elephant trunk for arch or proximal descending aneurysms.

Currently, our standard approach is one-stage robotic ligation and division of the ASA, followed by open supraclavicular SCT. Chart reviews were initially conducted in October 2024 and finalized in March 2025.

### Preoperative Evaluation

Patients referred to the aortic and thoracic clinics underwent a comprehensive evaluation, including history, physical examination, and cross-sectional imaging (eg, computed tomography, magnetic resonance imaging). The nature, onset, and progression of any esophageal or airway compressive symptoms were carefully assessed. Indications for surgical intervention for an ASA include long-standing and/or bothersome compressive symptoms. Contraindications for the robotic approach include the inability to tolerate single-lung ventilation, dense pleural adhesions, and a large aortic or Kommerell aneurysm.

### Surgical Techniques

The operative steps described here are for ARSA. The technique for ALSA is similar, with adjustments for laterality.

#### Robot-assisted division of ASA

**Figure 3:**
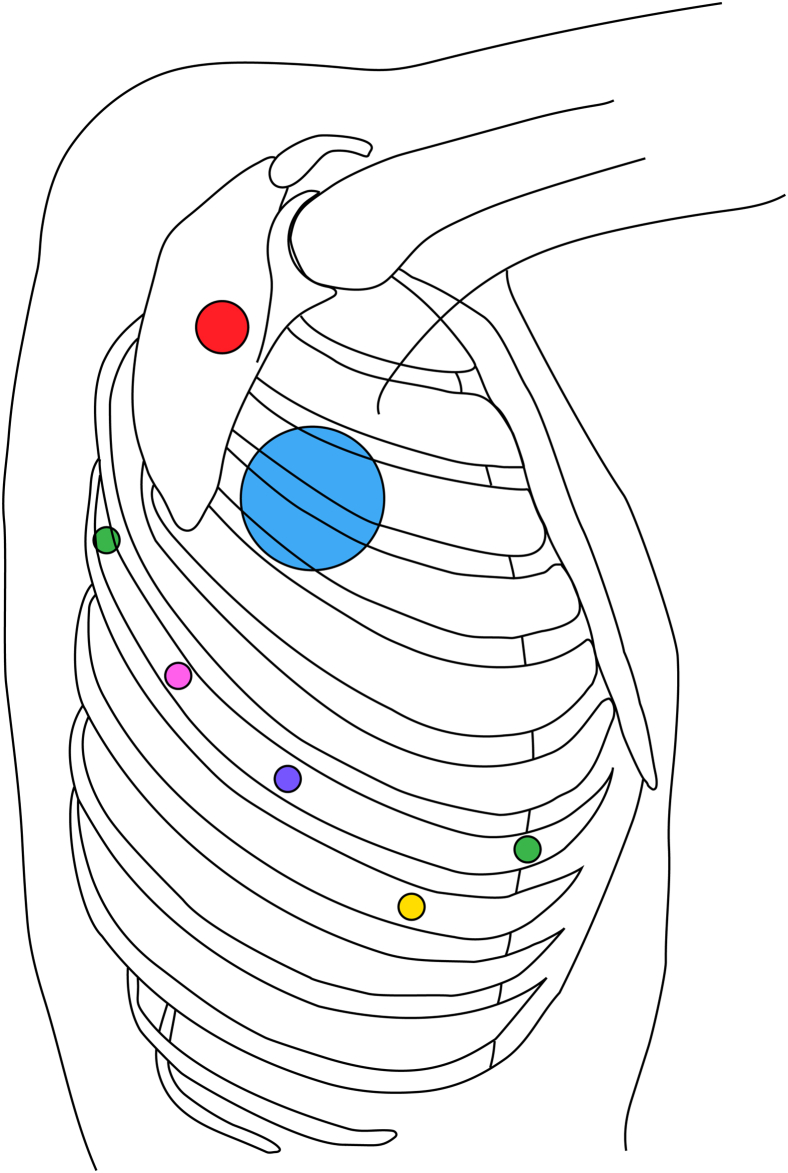
Standard port placement for robotic division of aberrant subclavian artery. *Purple*, robot camera port; *pink*, robot arm port, used for vascular stapler; *green*, robot arm ports; *yellow*, bedside assisting port; *red*, origin of aberrant right subclavian artery; *blue*, location of azygos vein. For an aberrant left subclavian artery, port placement is mirrored to the left hemithorax accordingly.

After induction of general anesthesia with a double-lumen endotracheal tube, the patient is positioned in the left lateral decubitus position (right lateral for ALSA) with the ipsilateral arm extended and the contralateral arm elevated. The Da Vinci Xi system is used, with standard port placement for thoracic robotic surgery (Figure 3). Five ports (4 robotic ports and 1 bedside assisting port) are placed within the right hemithorax (left hemithorax for ALSA). Once the ports are secured, the robot arms are docked. No anticoagulation is used for the intrathoracic portion of the procedure.

On entering the chest, the lung is retracted caudally to expose the upper mediastinum. The course of the retroesophageal ARSA is visualized at this stage. The pleura overlying the ARSA is incised and extended toward the thoracic inlet. Dissection is performed circumferentially around the ARSA from its aortic origin to its branching into the right vertebral and right internal mammary arteries. The mobilization allows the ARSA to be pulled into the neck during the second portion of the procedure. The ARSA is then divided and sealed at its takeoff using a robotic vascular stapler with a white load (open height, 2.5 mm; closed height, 1.0 mm). The esophagus and other mediastinal structures are carefully examined to ensure no further vascular compression or constriction is present. After ensuring hemostasis, the robotic instruments are removed, and the robot arms are undocked. A small chest tube is placed, the trocars are removed, and the port sites are closed following reinflation of the lung.

#### Supraclavicular SCT

The patient is repositioned supine on the operative table with both arms tucked. A 4- to 5-cm incision is made approximately 2 cm above the right clavicle (left clavicle for ALSA), centered between the 2 heads of the sternocleidomastoid muscle. The platysma muscle is divided, and dissection is carried down into the thoracic outlet. The right common carotid artery (left common carotid artery for ALSA) is identified and dissected out. The ARSA, which has been mobilized from the pleural space during the intrathoracic portion of the procedure, is then identified and brought up to the neck. Next, systemic heparinization is given to achieve an activated clotting time >250 seconds. Following clamping of the subclavian artery, the staple line is resected. The common carotid artery is clamped proximally and distally. A carotid arteriotomy is created for an end-to-side anastomosis with the subclavian artery.

On completion of the anastomosis, the proximal carotid clamp is removed first to allow flow into the subclavian artery, followed by the removal of the distal clamp. Doppler ultrasound is used to assess the flow in both the carotid artery distal to the anastomosis and the transposed subclavian artery. After confirming hemostasis, the incision is closed in layers.

### Postoperative Care

The patient is admitted to the cardiothoracic intensive care unit (ICU) for closed neurovascular monitoring. In addition to general postoperative care, attention is directed to changes in compressive symptoms, such as dysphagia and shortness of breath, as well as signs of hoarseness and Horner syndrome (eg, ptosis, miosis, anhidrosis). Routine esophagraphy or evaluation by speech and language therapy is not performed unless the patient has a history of significant or worsening dysphagia. The patient can be transferred out of the ICU as early as postoperative day 1 if there are no neurovascular deficits, no need for vasoactive infusions, and no respiratory concerns. The chest tube can be removed once the patient is tolerating a full diet without evidence of chyle leak. Most patients are discharged within 2 to 3 days.

The first postoperative check is within 2 to 4 weeks from surgery to assess wound and symptom improvement. Ongoing imaging surveillance for potential thoracic endovascular aortic repair (TEVAR) is performed at 3 months, 6 months, 1 year, and then annually thereafter to monitor for changes in the aorta and expansion of the ASA stump or KD (if present) by >2 cm.

### Variables and Data Analysis

Preoperative demographics, presentation, comorbidities, and anatomy of the aortic anomaly were assessed. Operative details and postoperative outcomes were evaluated. The dysphagia improvement scale was defined as 0, unchanged; 1, mildly improved; 2, moderately improved; and 3, significantly improved. Scoring was based on clinical documentation, including the patient's own words and clinician's descriptions. For example, “occasional choking” and “occasional sensation of difficulty swallowing” were categorized as mildly improved; “better but not perfect” was scored as moderately improved; and such descriptions as “no dysphagia,” “significantly improved,” “much improved,” and “improved” without mention of any residual symptoms were scored as significantly improved. The scale provided a relatively standardized assessment of subjective dysphagia improvement based on clinical notes.

Data collection and handling were performed with Microsoft Excel. Descriptive statistics, including mean ± SD, median (interquartile range [IQR]), and count (%), were calculated. The Shapiro-Wilk test was conducted using Python to assess the normality of continuous variables. Three-dimensional reconstructed illustrations were generated using 3D Slicer software.^10^

## Results

The 9 patients in the study cohort included 6 females (Table 1). The mean age was 49 ± 17.2 years, and mean body mass index was 30.5 ± 6. The most common presenting symptoms were dysphagia (n = 9; 100%), followed by dyspnea (n = 3; 33.3%) and cough (n = 3; 33.3%). Most patients had none or few preoperative comorbidities. Five patients had ALSA with a right-sided arch, and the other 4 patients had ARSA. KD was present in 7 patients, with an average diameter of 1.9 ± 0.7 cm. The average surveillance time prior to surgery was 4 ± 3.7 years.

**Table 1:** Summary of patient demographics, presentations, comorbidities, aberrant anatomy, operative details, and postoperative outcomes in those undergoing robotic division of ASA

Variable	Patient								
	1	2	3	4	5	6	7	8	9∗
Age, y	56	29	42	77	43	72	27	35	60
Sex	Female	Male	Female	Male	Female	Male	Female	Female	Female
BMI, kg/m^2^	23.6	30.1	26.1	29.6	25.4	43.1	31.7	26.8	38.0
Presentation	Dysphagia Globus Cough	Dysphagia	Dysphagia	Dysphagia Globus Shortness of breath Cough Hoarseness	Dysphagia Shortness of breath	Dysphagia Shortness of breath Wheezing	Dysphagia	Dysphagia	Dysphagia Cough
Preoperative comorbidities	Migraine Neuralgia Meningioma	None	HTN Former smoker	HTN HLD Afib Former smoker	Current smoker	HTN HLD CKD	HLD	OSA Migraine	HTN HLD CKD
Previous cardiac/aortic surgery	No	No	No	Yes	No	No	No	No	No
ASA	Right	Left	Right	Left	Right	Left	Left	Right	Left
Right-sided arch	No	Yes	No	Yes	No	Yes	Yes	No	Yes
KD	Yes, 1.2 cm	Yes, 2.1 cm	No	Yes, 3 cm	Yes, 1.7 cm	Yes, 3.2 cm	Yes, 1.9 cm	No	Yes, 1.7 cm
Surveillance time prior to surgery, y	3	4	1	10	2	12	6	6	<1
Open SCT	Right	Left	Right	No (previous CS bypass)	Right	Left	Left	Right	Left
ASA class	3	3	4	3	2	3	2	3	3
Operative time, min	198	196	116	120	169	243	164	177	160
Blood transfusion at any time	None	None	None	None	None	None	None	None	None
LOS, d	2	2	4	1	2	3	3	2	3
ICU LOS, d	2	<1	<1	0	<1	1	1	2	2
Stroke/TIA	No	No	No	No	No	No	No	No	No
Bleeding	No	No	No	No	No	No	No	No	No
Pneumothorax	No	No	No	No	No	No	No	No	No
Other complications	No	No	No	No	No	Hoarseness	No	Horner syndrome	No
Symptom improvement	Unknown	Unchanged	Mild	Significant	Significant	Significant	Significant	Moderate	Significant
Reintervention	No	No	No	No	No	No	No	No	No
TEVAR	No	No	No	Yes (for large DTA aneurysm and KD prior to division of ASA)	No	No	No	No	No
Mortality	No	No	No	No	No	No	No	No	No

*ASA*, Aberrant subclavian artery; *BMI*, body mass index; *HTN*, hypertension; *HLD*, hyperlipidemia; *OSA*, obstructive sleep apnea; *Afib*, atrial fibrillation; *CKD*, chronic kidney disease; *KD*, Kommerell diverticulum; *SCT*, subclavian-carotid transposition; *ASA*, American Society of Anesthesiologists; *LOS*, length of stay; *ICU*, intensive care unit; *TIA*, transient ischemic attack; *TEVAR*, thoracic endovascular aortic repair; *DTA*, descending thoracic aorta.

∗ This patient underwent the surgery 1 month prior to the data collection for this study.

Eight patients underwent concurrent SCT. One had prior TEVAR and carotid-subclavian bypass for large descending thoracic and Kommerell aneurysms 1 year before the resection of ASA. The mean operative time was 169 ± 37.1 minutes, and the mean hospital stay was 2 ± 0.8 days. No blood transfusions were needed for any patient at any time. One patient who did not have SCT was not admitted to the ICU postoperatively. For patients admitted to the ICU, the mean length of stay was 1.2 ± 0.6 days.

There was no postoperative stroke, bleeding, pneumothorax, chyle leak, or mortality. One patient developed hoarseness secondary to left vocal cord dysfunction, and another patient experienced transient postoperative Horner syndrome 1 week after discharge. Regarding dysphagia, 5 patients experienced significant improvement, 1 patient had moderate improvement, 1 had mild improvement, and 1 reported no change. The median dysphagia improvement score was 3 (IQR, 1.75-3), suggesting overall significant symptom relief. One patient was lost to follow-up, with no documentation regarding their symptoms. Excluding this patient, all other patients had stable postoperative imaging, and no reintervention was required during the surveillance period. The median time to most recent cardiac surgery follow-up was 94 days (IQR, 62-115 days).

## Discussion

The management of ASA has evolved significantly over time. Historically, open repair was the standard treatment, tailored to the patient's aberrant anatomy, associated arch pathology, and operative risk.^11^ Open approaches ranged from sternotomy and thoracotomy to cervicothoracic hybrid techniques, but these carried significant morbidity. The evolution of less invasive techniques aimed to reduce recovery time and perioperative complications. Over the past 2 decades, advances such as VATS, endovascular techniques, and hybrid approaches have led to improved outcomes in patients requiring ASA repair. More recently, the robotic-assisted approach has emerged as a promising alternative, offering greater visualization and precision for complex upper mediastinal dissections.

For ASA without aneurysmal changes, both a mediastinoscopy-assisted supraclavicular approach and VATS division of ASA with SCT have been used to avoid the need for conventional thoracotomy, improving postoperative recovery while reducing pain.^7^^,^^12^^,^^13^ Despite the former being proposed to improve exposure, the proximal dissection toward the origin of ASA remains challenging. In cases of significant bleeding, the mediastinoscopic approach to readily control the bleeding might not be feasible. In contrast, the thoracoscopic approach has been proven to further enhance surgical visualization and streamline dissection.^7^ Further refinement of ASA division at our institution is a robotic-assisted approach.

Additionally, an endovascular or hybrid approach is often reserved for patients with aneurysmal ASA/KD, but this approach poses challenges owing to anatomic prerequisites and the risks of worsening esophageal compression.^2^ Hybrid repair is performed by combining carotid-subclavian bypass and TEVAR with proximal ASA embolization or ligation^14^^,^^15^; however, these methods carry risks of ASA-esophageal fistulas and persistent retrograde flow.^16^ Given the elevated risks of endoleak and reintervention, long-term surveillance is necessary.^17^, ^18^, ^19^ Consequently, a total endovascular approach is used primarily in emergencies or high-risk patients with the goal of temporizing the risks of aneurysm rupture. For more complex cases, total arch replacement with frozen elephant trunk following debranching is indicated.^20^

**Figure 4:**
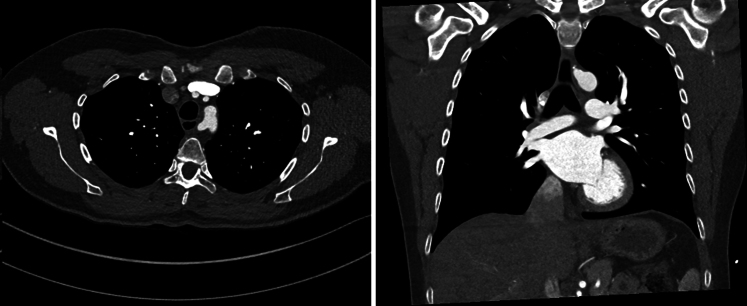
Contrast-enhanced computed tomography images demonstrating changes after robotic division of aberrant right subclavian artery. *Left*, axial view; *right*, coronal view.

**Figure 5:**
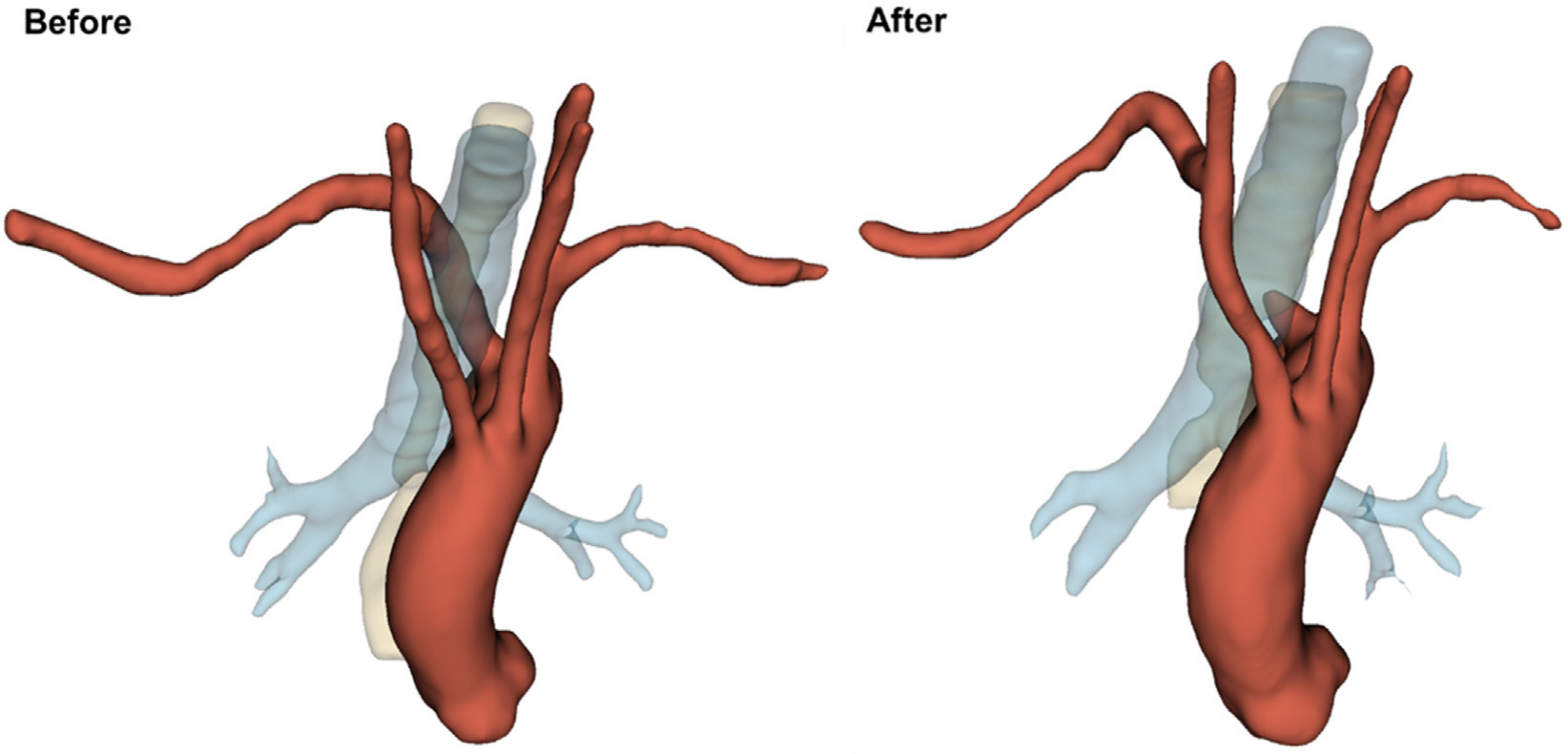
Three-dimensional illustrations depicting the course of the aberrant right subclavian artery before and after surgery. *Red*, aorta and its branches; *blue*, trachea and bronchi; *yellow*, esophagus.

In the present study, the most common indications were dysphagia and dyspnea, consistent with previous studies.^1^^,^^5^ Although generally rarer than ARSA, there were more ALSA patients (n = 5; 55.6%) in this cohort. The prevalence of KD was 77.8%, higher than the 20% to 60% reported by previous studies.^6^ Our results demonstrate excellent postoperative outcomes, including a very short length of stay, significant dysphagia relief in most patients, absence of short-term reintervention, and lack of operative mortality. We have defined a scale to assess dysphagia improvement through a retrospective review of clinical notes. Although not perfect, this scale provides a useful way to monitor our patient's postoperative progression. Five patients had fully recovered and were free of swallowing difficulty at their most recent cardiac surgery follow-up. In 3 patients, the incomplete resolution of dysphagia was believed to have resulted from incomplete surgical mobilization of the esophagus following relief of compression. We increased emphasis on this aspect of the surgery after some patients with residual symptoms were recognized. One patient experienced a delayed onset of transient Horner syndrome, likely due to postoperative edema and inflammation or to direct trauma during surgical manipulation, affecting the cervical sympathetic chain. Of note, others have reported a 4% incidence of postoperative Horner syndrome associated with a supraclavicular approach to ASA bypass or transposition.^21^ In addition, 1 patient with ALSA and a 3.2-cm KD developed mild dysphonia and hoarseness, possibly caused by recurrent (or variant) laryngeal nerve injury. There was no sign of recovery after 2 months of observation, prompting further soft tissue filler (ie, carboxymethylcellulose) injection to the vocal cord by Otolaryngology. One patient was lost to follow-up after the first postoperative appointment. The median time from surgery to the most recent follow-up was 94 days, and the longest time was 443 days. All other patients with available imaging data had stable ASA stump and aorta sizes (Figure 4). Figure 5 illustrates the course of ARSA before and after surgery.

Unlike our one-stage approach, 2 other reports of robotic approaches to ASA used a two-stage strategy involving a carotid-subclavian bypass and robotic division of the ASA at a later date.^8^^,^^9^ However, the use of synthetic grafts for carotid-subclavian bypass carries the potential of graft infection and thrombosis, while transposing the native subclavian artery theoretically has a lower risk. In the present study, no postoperative infection or anastomotic complications were observed. Our one-stage approach shortened the hospital stay dramatically, with an average of 2 to 3 days, with higher patient satisfaction.

Although the robotic results appeared similar to the VATS outcomes reported previously by our team, the robotic approach offers surgeons enhanced precision and dexterity, which may further reduce stress on the surgeon and facilitate dissection. The 4 robotic ports are placed in the same intercostal space, sharing the same nerve distribution to optimize postoperative pain control. Since the latest Da Vinci 5 is enabled with tactile feedback and improved 3-dimensional vision, dissection in the upper mediastinum around the great vessels and mobilization of ASA potentially will be safer and faster. The robotic, intrathoracic portion of the procedure is performed primarily by thoracic surgeons, and the supraclavicular, extrathoracic portion is subsequently performed by aortic surgeons. To ligate and divide the proximal subclavian artery, a robotic vascular stapler is used. In the event of massive intraoperative bleeding necessitating open conversion, our contingency plan is to proceed with a thoracotomy using the current patient positioning to promptly control the bleeding, although our team has not encountered such a complication. Similar to the VATS approach, the robotic technique is considered appropriate for symptomatic patients with nonaneurysmal ASA and KD.^7^ Although no specific intraoperative maneuvers were implemented to prevent the formation of pseudoaneurysm or arterial-esophageal fistula, continued postoperative imaging surveillance is needed in all patients to monitor for degeneration of the remaining subclavian stump, aortic arch, and descending thoracic aorta, as well as other potential complications. In cases of later aneurysm degeneration, an endovascular approach is used to exclude the ASA/KD remnant or thoracic aorta. Prophylactic TEVAR exclusion is not routinely performed at our institution.

Given the very recent adoption of the robotic approach, no mid-term or long-term data are available. The most recent patient underwent surgery just 1 month prior to the data collection for this report, so no postoperative imaging was available. However, significant improvement in dysphagia was reported during the first postoperative clinic visit. As the robotic approach shows promising early outcomes, ongoing surveillance and further follow-up are necessary to evaluate for the long-term outcomes, including symptom relief, durability of ASA stump management by the vascular stapler, and the potential need for reinterventions.

### Limitations

This is a retrospective single-center study with a small sample size owing to the rare prevalence of ASA, and thus no comparative statistical analysis was performed. The follow-up period is relatively short owing to the recent use of the roboticic technique, with the most recent patient undergoing surgery just 1 month before data collection. The mid-term and long-term durability of this robotic approach remains unknown. Given the rarity of cases, no direct comparison between different techniques was conducted at our institution.

The dysphagia improvement scale is based on retrospective chart reviews, which may introduce reporting and observer bias, although patients’ own statements are typically documented in the clinical notes. However, this study design did not include patient follow-up through phone calls or additional surveys to assess changes in symptoms.

## Conclusions

The one-stage robotic division of the ASA and supraclavicular SCT is a safe, minimally invasive alternative for treating dysphagia lusoria, with no reported postoperative stroke or mortality. Despite the small sample size and short follow-up, this approach shows promising dysphagia relief and great patient satisfaction. Long-term surveillance is needed to assess durability and monitor for potential aneurysm degeneration.

### Webcast

You can watch a Webcast of this AATS meeting presentation by going to: https://www.aats.org/resources/outcomes-of-robotic-approach-t-10010.

**Figure:**
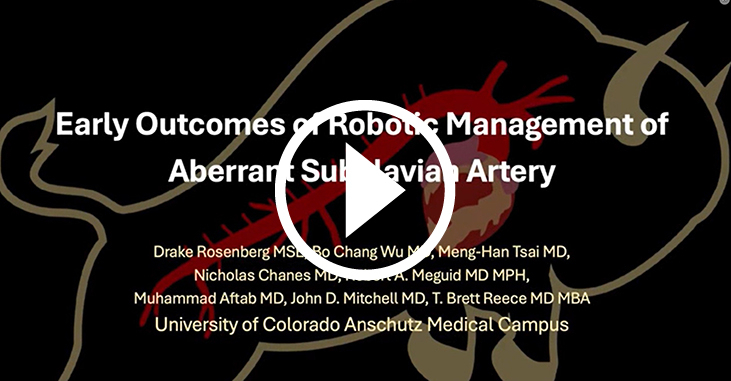

## Conflict of Interest Statement

The authors reported no conflicts of interest.

The *Journal* policy requires editors and reviewers to disclose conflicts of interest and to decline handling or reviewing manuscripts for which they may have a conflict of interest. The editors and reviewers of this article have no conflicts of interest.

## References

[1] Jahangeer S., Bashir M., Harky A., Yap J. (2018). Aberrant subclavian: new face of an old disease. J Vis Surg.

[2] Yang C., Shu C., Li M., Li Q., Kopp R. (2012). Aberrant subclavian artery pathologies and Kommerell's diverticulum: a review and analysis of published endovascular/hybrid treatment options. J Endovasc Ther.

[3] Hunauld P.M. (1735). Examen de quelques parties d'un singe. Hist Acad Roy Sci.

[4] Bayford D. (1787).

[5] Polguj M., Chrzanowski Ł., Kasprzak J.D., Stefánczyk L., Topol M., Majos A. (2014). The aberrant right subclavian artery (arteria lusoria): the morphological and clinical aspects of one of the most important variations--a systematic study of 141 reports. ScientificWorldJournal.

[6] Czerny M., Grabenwöger M., Berger T., Authors/Task Force Members (2024). EACTS/STS guidelines for diagnosing and treating acute and chronic syndromes of the aortic organ. Ann Thorac Surg.

[7] Ghincea C.V., Ikeno Y., Weyant M.J., Mitchell J.D., Aftab M., Reece T.B. (2020). Right thoracoscopic aberrant right subclavian artery division and subclavian-carotid transposition. Ann Thorac Surg.

[8] La Regina D., Prouse G., Mongelli F., Pini R. (2020). Two-step treatment of dysphagia lusoria: robotic-assisted resection of aberrant right subclavian artery following aortic debranching. Eur J Cardiothorac Surg.

[9] Meredith L.T., Isch E.L., Ali M.I., Nooromid M.J., Okusanya O.T. (2024). Technical considerations in robotic aberrant right subclavian artery resection for dysphagia lusoria. J Vasc Surg Cases Innov Tech.

[10] Fedorov A., Beichel R., Kalpathy-Cramer J. (2012). 3D slicer as an image computing platform for the quantitative imaging network. Magn Reson Imaging.

[11] Kieffer E., Bahnini A., Koskas F. (1994). Aberrant subclavian artery: surgical treatment in thirty-three adult patients. J Vasc Surg.

[12] Moorjani N., Mohsen N., Boateng P. (2010). Mediastinoscopy-assisted ligation of an aberrant right subclavian artery through a supraclavicular approach. J Thorac Cardiovasc Surg.

[13] Fukuhara S., Patton B., Yun J., Bernik T. (2013). A novel method for the treatment of dysphagia lusoria due to aberrant right subclavian artery. Interact Cardiovasc Thorac Surg.

[14] Frigatti P., Grego F., Deriu G.P., Lepidi S. (2009). Hybrid endovascular treatment of aneurysm degeneration in a rare right-aortic arch anomaly with Kommerell diverticulum. J Vasc Surg.

[15] Bloom J.P., Attia R.Q., Sundt T.M. (2021). Outcomes of open and endovascular repair of Kommerell diverticulum. Eur J Cardiothorac Surg.

[16] Verzini F., Isernia G., Simonte G., De Rango P., Cao P., Italian AARSA Collaborative Group (2015). Results of aberrant right subclavian artery aneurysm repair. J Vasc Surg.

[17] Gray S.E., Scali S.T., Feezor R.J. (2020). Safety and efficacy of a hybrid approach for repair of complicated aberrant subclavian arteries. J Vasc Surg.

[18] Loschi D., Santoro A., Rinaldi E., Bilman V., Chiesa R., Melissano G. (2023). A systematic review of open, hybrid, and endovascular repair of aberrant subclavian artery and Kommerell's diverticulum treatment. J Vasc Surg.

[19] Fukuhara S., Ahmed Y., Shiomi S. (2022). Aberrant subclavian arteries and associated Kommerell diverticulum: endovascular vs open repair. Ann Thorac Surg.

[20] Li J.R., Ma W.G., Chen Y. (2020). Total arch replacement and frozen elephant trunk for aortic dissection in aberrant right subclavian artery. Eur J Cardiothorac Surg.

[21] Tallarita T., Rogers R.T., Bower T.C. (2023). Characterization and surgical management of aberrant subclavian arteries. J Vasc Surg.

